# The modified Glasgow Prognostic Score indicates an increased risk of anastomotic leakage after anterior resection for rectal cancer

**DOI:** 10.1007/s00384-023-04496-5

**Published:** 2023-07-20

**Authors:** Parisa Golshani, Jennifer Park, Jenny Häggström, Josefin Segelman, Peter Matthiessen, Marie-Louise Lydrup, Martin Rutegård, on behalf of the RectoLeak study group, Anders Gerdin, Olle Sjöström, Maria Staffan, Staffan Jangmalm, Hanna Royson, Konstantinos Tsimogiannis, Kajsa Anderin, Jonas Nygren, Jennie Hurtig

**Affiliations:** 1 Department of Surgery, Regional Council of Gävleborg, Gävle, Sweden; 2 Department of Surgery, SSORG - Scandinavian Surgical Outcomes Research Group, Institute of Clinical Sciences, Sahlgrenska Academy, University of Gothenburg, Gothenburg, Sweden; 3 Department of Statistics, Umeå School of Business, Economics and Statistics, Umeå University, Umeå, Sweden; 4 Department of Molecular Medicine and Surgery, Karolinska Institutet, and Ersta Hospital, Stockholm, Sweden; 5 Department of Surgery, Faculty of Medicine and Health, Örebro University, Örebro, Sweden; 6 Department of Surgery, Skåne University Hospital, Malmö, Lund University, Lund, Sweden; 7 Department of Surgical and Perioperative Sciences, Surgery, Umeå University, SE-901 85 Umeå, Sweden; 8 Wallenberg Centre for Molecular Medicine, Umeå University, Umeå, Sweden; 9 Department of Radiation Sciences, Umeå University, Umeå, Sweden; 10 Department of Surgery, Falun Hospital, Falun, Sweden; 11 Department of Surgery, Växjö Hospital, Växjö, Sweden; 12 Department of Surgical Sciences, Uppsala University, Uppsala, Sweden; 13 Department of Surgery, Sunderby Hospital, Luleå, Sweden

**Keywords:** Anastomotic leak, Colorectal, Inflammation

## Abstract

**Background:**

Preoperative inflammation might cause and also be a marker for anastomotic leakage after anterior resection for rectal cancer. Available biomarker indices such as the modified Glasgow Prognostic Score (mGPS) or the C-reactive protein-to-albumin ratio (CAR) may be clinically useful for leakage assessment.

**Methods:**

Patients who underwent anterior resection for rectal cancer during 2014–2018 from a multicentre retrospective cohort were included. Data from the Swedish Colorectal Cancer registry and chart review at each hospital were collected. In a subset of patients, preoperative laboratory assessments were available, constituting the exposures mGPS and CAR. Anastomotic leakage within 12 months was the outcome. Causally oriented analyses were conducted with adjustment for confounding, as well as predictive models.

**Results:**

A total of 418 patients were eligible for analysis. Most patients had mGPS = 0 (84.7%), while mGPS = 1 (10.8%) and mGPS = 2 (4.5%) were less common. mGPS = 2 (OR: 4.11; 95% CI: 1.69–10.03) seemed to confer anastomotic leakage, while this was not seen for mGPS = 1 (OR 1.09; 95% CI: 0.53–2.25). A cut off point of CAR > 0.36 might be indicative of leakage (OR 2.25; 95% CI: 1.21–4.19). Predictive modelling using mGPS rendered an area-under-the-curve of 0.73 (95% CI: 0.67–0.79) at most.

**Discussion:**

Preoperative inflammation seems to be involved in the development of anastomotic leakage after anterior resection for cancer. Inclusion into prediction models did not result in accurate leakage prediction, but high degrees of systemic inflammation might still be important in clinical decision-making.

**Supplementary Information:**

The online version contains supplementary material available at 10.1007/s00384-023-04496-5.

## Introduction

Anastomotic leakage after anterior resection for rectal cancer affects 10–20% of patients [1, 2], resulting in bowel dysfunction [3], permanent stoma and increased mortality [4]. While the pathophysiology of anastomotic leakage remains to be fully elucidated, there are established risk factors including male sex, low anastomosis, radiotherapy, advanced comorbidity [5] and smoking [6].

Previous research suggests associations with preoperative systemic inflammation, as measured by e.g. C-reactive protein (CRP) and albumin, and anastomotic leakage after gastrointestinal surgery [7–9]. However, few studies have been conducted on preoperative inflammation and the risk of anastomotic leakage in anterior resection for rectal cancer [10]. A well-known index of inflammation, with prognostic value for survival, is the modified Glasgow Prognostic Score (mGPS) [11]; another measure, increasingly used in research, is the CRP-to-albumin ratio (CAR) [8, 9, 12, 13]. As both measures are based on routine blood samples, they are easy to use and readily available markers of preoperative inflammation. If any such a measure can be validated as being predictive of anastomotic leakage, this could aid patients and surgeons in clinical decision-making, potentially reducing the use of temporary stomas in low-risk patients and avoiding an anastomosis altogether in high-risk patients.

In this multicentre retrospective study, our primary aim was to investigate whether preoperative mGPS or CAR are useful predictors of anastomotic leakage after anterior resection for rectal cancer. Our secondary purpose was to incorporate mGPS and CAR into predictive models for leakage and evaluate their accuracy.

## Method

### Study design

This is a retrospective multicentre cohort with 11 participating Swedish centres. Patients who underwent anterior resection from 2014 to 2018 were identified at each hospital. Exclusion criteria comprised other indications than rectal cancer and missing preoperative CRP and albumin. Data such as previous medical and surgical history, standing medication and surgical techniques were collected at each centre through chart review. Postoperative data comprising short- and long-term outcomes, including anastomotic leakage up to 12 months after index surgery, were also registered. The resulting information was entered in the RedCap system (Vanderbilt University, Tennessee, USA), which is a web-based application to capture data for clinical research.

Further data were retrieved through the Swedish Colorectal Cancer Registry (SCRCR). The SCRCR is a national registry with data from all hospitals operating patients with colorectal cancer in Sweden. It has been validated with excellent results, reaching a completeness of inclusion in 99% [14]. The RedCap database and SCRCR data were linked to ensure all patients registered in RedCap had surgery due to rectal cancer. Furthermore, data such as demographics, preoperative variables, tumour stage and height, neoadjuvant treatment and some perioperative data were added from the SCRCR.

### Exposure

The primary exposure was the mGPS, an index based on combining albumin plasma levels and elevated CRP levels. The prognostic score has three categories: 0, 1 or 2. Patients with CRP ≤ 10 mg/l and any albumin level are allocated a score of 0, while patients with CRP > 10 mg/l and albumin levels ≥ 35 g/l are scored as 1; patients with CRP > 10 mg/l and albumin < 35 g/l are scored as 2 [11]. The secondary exposure was the CAR. Both measures were calculated using preoperative blood samples ascertained by chart review, where these samples were taken within 30 days prior to surgery.

### Outcome

The primary outcome was anastomotic leakage within 12 months after anterior resection. The definition of anastomotic leakage was in accordance with the international consensus definition from the International Study Group of Rectal Cancer [15]. Anastomotic leakage was thus defined as a communication between the intra- and extra-luminal compartments owing to a defect of the integrity of the intestinal wall at the level of anastomosis or suture/staple line, as well as an abscess in the proximity of the anastomosis. Anastomotic leakage was further divided into grades A, B and C. Grade A was defined as leakage requiring no active therapeutic intervention, corresponding to a radiological leakage with minimal clinical signs. Grade B was defined as leakage requiring an active therapeutic intervention, barring a laparotomy. Grade C was defined as leakage requiring laparotomy; in this study, relaparoscopy also rendered a grade C. Anastomotic leakage may also be classified into early or late, consistent with a diagnosis within or after 30 days, respectively [2]. In the SCRCR there is no formal definition of anastomotic leakage and it is only mandatory to register leakage within the first 30 days after primary surgery, leading to a potential underreporting of anastomotic leakage [16]. Using chart review instead in the current study, detailed data were collected on anastomotic leakage and subsequent management within a year from the primary surgery.

### Statistical analysis

Continuous variables were reported using the median along with interquartile range (IQR), while categorical variables were reported using numbers and percentages. Fisher´s exact test was applied to test for associations in selected comparisons of categorical variables.

**Fig. 1:**
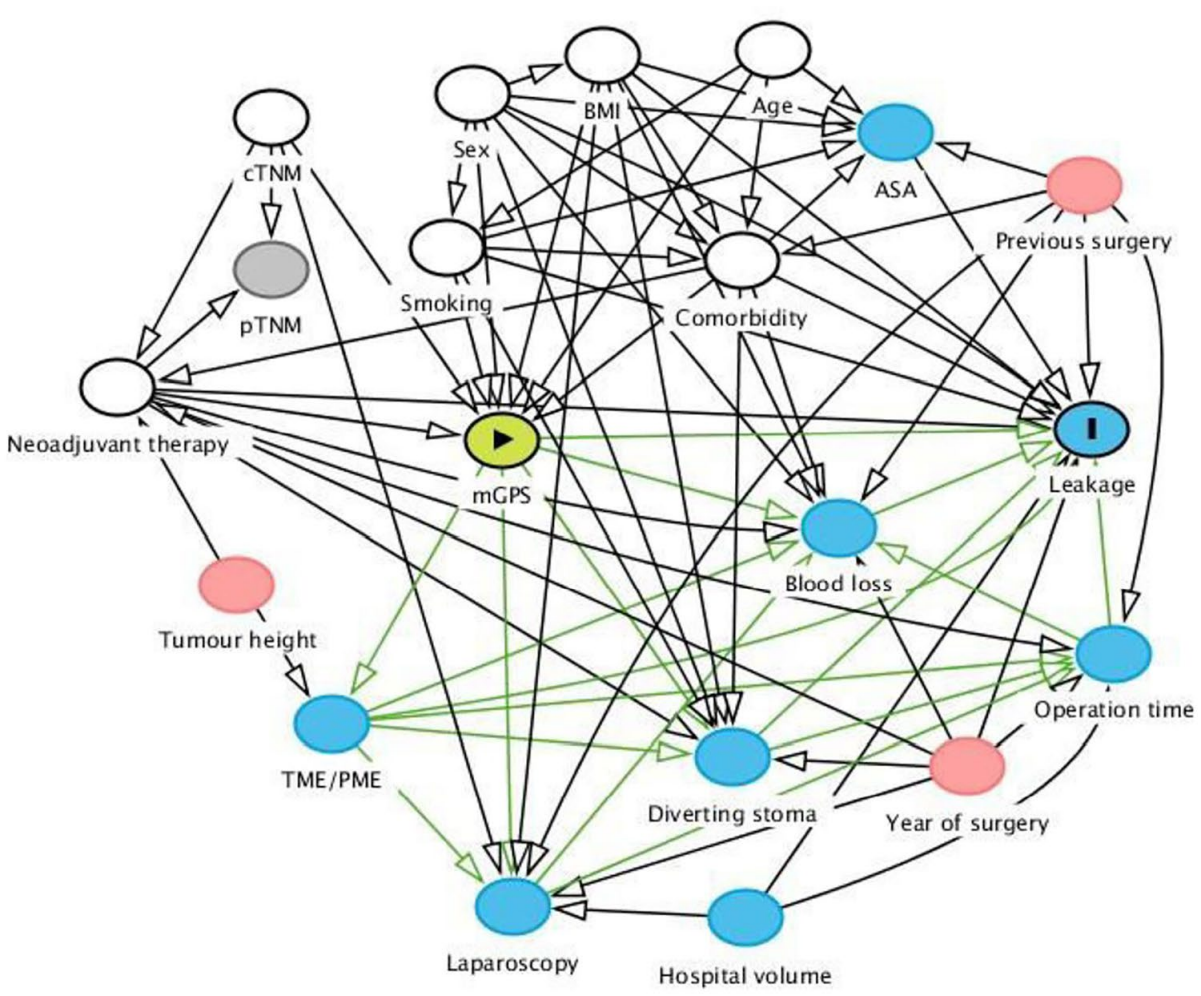
Directed acyclic graph picturing the assumed relationship between different variables potentially involved in the development of anastomotic leakage. Modified Glasgow Prognostic Score (mGPS) indicates exposure and anastomotic leakage is outcome; exposure is replaced with C-reactive protein-to-albumin ratio in the secondary analysis. A minimal adjustment set to derive a total effect on the outcome from exposure consisted of variables such as age, body mass index (BMI), sex, clinical tumour stage (cTNM), Charlson Comorbidity Index (CCI), smoking, and neoadjuvant therapy. TME/PME = total/partial mesorectal excision; ASA = American Society of Anesthesiologists’ fitness grade

A directed acyclic graph [17] is presented in Fig. 1. The graph shows the proposed causes and effects that are involved in the development of anastomotic leakage. The following covariates were selected in order to have minimally sufficient adjustment set: age (years; continuous), body mass index (BMI; kg/m^2^; continuous), sex (male or female), clinical T stage (T1-2, T3, T4 or TX), clinical N stage (N0, N1-2 or NX), clinical M stage (M0 or M1), Charlson Comorbidity Index (CCI; 0, 1, 2 or ≥ 3), current smoking (no or yes), neoadjuvant therapy (none, radiotherapy or chemoradiotherapy).

To estimate the effects of preoperative mGPS > 0 on anastomotic leakage within 12 months we used targeted maximum likelihood estimation [18, 19]. This is a two-step procedure designed to obtain an optimal bias-variance trade-off when estimating the effect of an exposure on outcome. The initial outcome and propensity score models were fitted using lasso regression [20], and the set of covariates described above was included in the models as potential confounders. The results are expressed as odds ratios (ORs), together with their 95% confidence intervals (CIs), calculated with influence-curve based standard errors [18].

Additionally, logistic regression analysis was performed to assess independent associations of mGPS with anastomotic leakage when including other relevant predictors in a multivariable prediction model. In addition to the covariates outlined above, the prediction model included the American Society of Anesthesiologists’ (ASA) fitness grade, extent of mesorectal excision (total [TME] or partial [PME]), defunctioning stoma (no or yes), previous surgery (no or yes), hospital volume and surgical technique (open or minimally invasive [conventional laparoscopy or robotically assisted laparoscopy]). A reduced model including only the subset of predictors selected by lasso regression was also fitted.

Analogous analyses as described for mGPS were also conducted with a categorized CAR as exposure. CAR was categorized by estimating the optimal location and number of cut-off points [18] for a continuous variable included in a pre-specified logistic regression model [21, 22]. The cut-off points were selected as those maximizing the area under the receiver operation characteristic curve. The pre-specified model used here included all predictors.

Multivariate imputation by chained equations was used to handle missing values [23] and the estimates from 10 imputed data sets were pooled according to Rubin’s rules [24]. All analyses were performed using R 4.2.1 statistical software [25]. The R-packages CatPredi [26], tmle [27], glmnet [28] and mice [29] were used for categorization, analysis and imputation.

## Results

### Patients

**Fig. 2:**
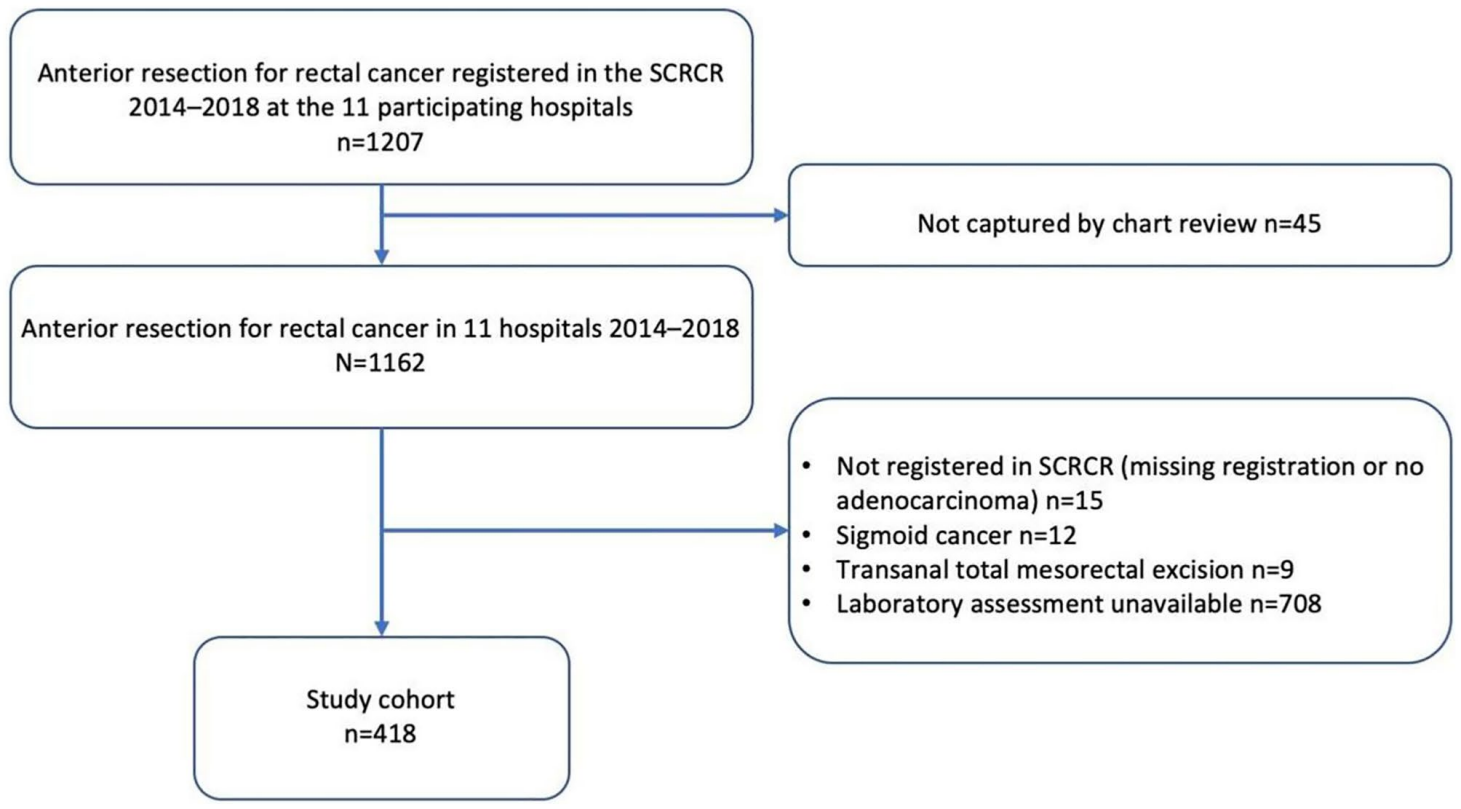
Study flowchart. SCRCR = Swedish Colorectal Cancer Registry

The original cohort included 1,126 eligible patients, while 708 patients were excluded due to unavailable data on preoperative CRP and/or albumin. After exclusion, 418 patients remained for analysis and a study flowchart is presented in Fig. 2.

Clinical and demographic variables are presented in Table 1. Most patients had mGPS = 0 (N: 354; 84.7%), while mGPS = 1 (N: 45; 10.8%) and mGPS = 2 (N: 19; 4.5%) were progressively rarer. While higher age, male sex, advanced comorbidity and current smoking were more common in the latter groups, BMI was similar across groups. Notably, patients with higher mGPS scores had more advanced tumours and subsequently received neoadjuvant therapy to a higher degree. Open surgery was also more common in these groups, while there were no apparent differences in type of mesorectal excision employed nor the use of defunctioning stoma. Duration of operation was longer and intraoperative bleeding was higher in the group with the highest mGPS score.

**Table 1:** Clinical and demographic data for *n* = 418 patients (patients with missing on preoperative C-reactive protein and preoperative albumin are excluded; patients with missing covariate values are included), stratified by the modified Glasgow Prognostic Score (mGPS)

	mGPS = 0 (N = 354)	mGPS = 1 (N = 45)	mGPS = 2 (N = 19)	Overall (N = 418)
Age (years)				
Median (IQR)	65.8 (58–72)	70.0 (62–75)	70.0 (63–72.5)	66.0 (59–72)
Sex				
Female	141 (39.8%)	17 (37.8%)	5 (26.3%)	163 (39.0%)
Male	213 (60.2%)	28 (62.2%)	14 (73.7%)	255 (61.0%)
Charlson Comorbidity Index				
0	253 (71.5%)	28 (62.2%)	11 (57.9%)	292 (69.9%)
1	57 (16.1%)	9 (20.0%)	5 (26.3%)	71 (17.0%)
2	28 (7.9%)	5 (11.1%)	1 (5.3%)	34 (8.1%)
≥ 3	16 (4.5%)	3 (6.7%)	2 (10.5%)	21 (5.0%)
ASA fitness grade				
I	83 (23.4%)	6 (13.3%)	1 (5.3%)	90 (21.5%)
II	220 (62.1%)	33 (73.3%)	10 (52.6%)	263 (62.9%)
III–V	48 (13.6%)	5 (11.1%)	8 (42.1%)	61 (14.6%)
Missing	3 (0.8%)	1 (2.2%)	0 (0%)	4 (1.0%)
Smoker				
No	314 (88.7%)	37 (82.2%)	14 (73.7%)	365 (87.3%)
Yes	22 (6.2%)	4 (8.9%)	2 (10.5%)	28 (6.7%)
Missing	18 (5.1%)	4 (8.9%)	3 (15.8%)	25 (6.0%)
Body mass index (kg/m^2^)				
Median (IQR)	25 (23–28)	25 (24–27)	26 (24–28)	25 (23–28)
Missing	6 (2%)	0 (0%)	0 (0%)	6 (1%)
Tumour height (cm)				
Median (IQR)	10 (9–12)	10 (9–12)	12 (10–13)	10 (9–12)
Missing	0 (0%)	1 (2%)	0 (0%)	1 (0%)
Clinical T stage				
cT1-2	141 (39.8%)	15 (33.3%)	2 (10.5%)	158 (37.8%)
cT3	175 (49.4%)	22 (48.9%)	13 (68.4%)	210 (50.2%)
cT4	27 (7.6%)	7 (15.6%)	4 (21.1%)	38 (9.1%)
cTX	11 (3.1%)	1 (2.2%)	0 (0%)	12 (2.9%)
Clinical N stage				
cN0	180 (50.8%)	16 (35.6%)	6 (31.6%)	202 (48.3%)
cN1-2	162 (45.8%)	29 (64.4%)	12 (63.2%)	203 (48.6%)
cNX	12 (3.4%)	0 (0%)	1 (5.3%)	13 (3.1%)
Clinical M stage				
cM0	335 (94.6%)	43 (95.6%)	18 (94.7%)	396 (94.7%)
cM1	19 (5.4%)	2 (4.4%)	1 (5.3%)	22 (5.3%)
Neoadjuvant therapy				
No	176 (49.7%)	18 (40.0%)	6 (31.6%)	200 (47.8%)
Radiotherapy	134 (37.9%)	18 (40.0%)	10 (52.6%)	162 (38.8%)
Radiochemotherapy	44 (12.4%)	9 (20.0%)	3 (15.8%)	56 (13.4%)
Surgical technique				
Open	125 (35.3%)	17 (37.8%)	10 (52.6%)	152 (36.4%)
Minimally invasive	229 (64.7%)	28 (62.2%)	9 (47.4%)	266 (63.6%)
Type of mesorectal excision				
Total	271 (76.6%)	31 (68.9%)	15 (78.9%)	317 (75.8%)
Partial	83 (23.4%)	14 (31.1%)	4 (21.1%)	101 (24.2%)
Defunctioning stoma				
No	68 (19.2%)	9 (20.0%)	4 (21.1%)	81 (19.4%)
Yes	286 (80.8%)	36 (80.0%)	15 (78.9%)	337 (80.6%)
Operation time				
Median (IQR)	290 (213–377)	260 (195–359)	326 (219–397)	289 (210–377)
Missing	2 (1%)	0 (0%)	0 (0%)	2 (1%)
Perioperative bleeding (ml)				
Median (IQR)	100 (50–313)	150 (50–350)	300 (100–950)	100 (50–350)
Missing	10 (3%)	0 (0%)	0 (0%)	10 (2%)

*IQR* interquartile range, *ASA* American Society of Anesthesiologists

### Modified Glasgow Prognostic Score and anastomotic leakage

In the analysed cohort, 74 patients (17.7%) had anastomotic leakage within 12 months (55 leaks occurred within 30 days, while 19 patients had late leakage). Of these, 10 were grade A, 42 were grade B, and 22 were grade C leaks. Patients with mGPS = 0 had a leak rate of 15.8% (n: 56), mGPS = 1 22.2% (n: 10) and mGPS = 2 42.1% (n: 8); Fisher’s exact test, p = 0.012. Table 2 demonstrates the average causal effect of preoperative mGPS > 0 on anastomotic leakage within 12 months with an OR of 1.09 (95% CI: 0.53–2.25) for mGPS = 1 and OR 4.11 (95% CI: 1.69–10.03) for mGPS = 2.

**Table 2:** Average causal effect of preoperative modified Glasgow Prognostic Score (mGPS) and anastomotic leakage within 12 months, estimated by targeted maximum likelihood estimation, yielding odds ratios (ORs) with 95% confidence intervals (CIs)

**Exposure**	**Leak rate (%)**	OR (95% CI)*
**mGPS = 0**	56 (15.8)	1.00 (reference)
**mGPS = 1**	10 (22.2)	1.09 (0.53–2.25)
**mGPS = 2**	8 (42.1)	4.11 (1.69–10.03)

*Covariates included in the lasso logistic regression models: Age, Body Mass Index, sex, clinical T stage, clinical N stage, clinical M stage, Charlson comorbidity index group, current smoking status, neoadjuvant therapy

### CRP-to-albumin ratio and anastomotic leakage

Using the genetic algorithm [18], cut-off point optimization was performed on each imputed data set and the average of the estimated optimal cut-off points was used for categorizing CAR into ≤ 0.36 and > 0.36. Patients with CAR ≤ 0.36 had a leak rate of 15.6% (n: 57), while patients with CAR > 0.36 experienced a leak frequency of 32.1% (n: 17); Fishers’s exact test, p = 0.006 (Table S1). The average causal effect of preoperative CAR > 0.36 on anastomotic leakage, in comparison to CAR ≤ 0.36, was estimated at an OR of 2.25 (95% CI: 1.21–4.19).

### Prediction modelling

#### mGPS

From the logistic regression model including all potential predictors, we found that mGPS = 2 (OR: 2.98; 95% CI: 1.01–8.82) had a stronger association to anastomotic leakage as compared to mGPS = 1 (OR: 1.42; 95% CI: 0.62–3.21). The reduced prediction model, including only predictors selected by lasso regression, showed similar results (Table S2). The full model resulted in an AUC of 0.73 (95% CI: 0.67–0.79), while the reduced model showed an AUC of 0.72 (95% CI: 0.66–0.79).

#### CAR

In both the full and reduced prediction models, CAR > 0.36 was found to be significantly associated with anastomotic leakage. As seen in Table S3, the full model resulted in an OR of 2.41 (95% CI: 1.16–5.00), and the reduced model displayed an OR of 2.32 (95% CI: 1.13–4.78). These models resulted in AUC 0.73 (95% CI: 0.66–0.79) and AUC 0.72 (95% CI: 0.66–0.79), respectively.

## Discussion

### Main results

In this multicentre study, the highest degree of preoperative inflammation, mGPS = 2, seemed to substantially increase the risk of anastomotic leakage within 12 months after anterior resection for rectal cancer, as compared to patients with mGPS = 0 or mGPS = 1. Similarly, using an internally derived cut-off, patients with a CAR > 0.36 were affected with a higher risk of leakage.

Incorporating the mGPS score and the CAR into prediction models for anastomotic leakage resulted in an only fairly performing model, particularly as the sample size did not permit internal validation, suggesting that more data are needed to accurately predict leakage after anterior resection for rectal cancer.

### Strengths and weaknesses

The cohort is not truly population-based, although both university hospitals and county hospitals from different geographical regions throughout Sweden provided data. At each centre, chart review was compared to SCRCR data, minimising misclassifications. An advantage of the current report is the relatively short study period of five years, which ensures consistent management and surgical technique within centres. Moreover, the 12-month follow-up through chart review, instead of a conventional time frame of 30 days as captured by most registry studies [14, 16], means that more anastomotic leaks were discovered, as up to one third of the leaks occur 30 days after surgery [2]. Weaknesses of this study include the retrospective design, where in particular a high degree of exclusion due to lack of laboratory assessment is a threat to the validity of the study; this might introduce selection bias, especially as some centres had a more systematic approach of assessing preoperative bloods than others. Anecdotally, some of the missing samples were due to long waiting times to surgery, where the sampling was done outside of the allowed 30-day window. This also translated into a relatively small analytical sample, as evidenced by the wide confidence intervals. Although the use of directed acyclic graphs enabled consideration of confounding, residual confounding from unobserved covariates and misclassification are limitations common to any retrospective study. For example, there was no data concerning weight change and nutritional therapy, which might have influenced the results.

### Literature review

A recently published large multicentre cohort identified low preoperative protein levels as an independent risk factor for anastomotic leakage after subtotal and total colectomy with ileorectal and ileosigmoid anastomosis in patients with colitis, polyposis disease and obstructed or synchronous colorectal cancer [30]. Although this patient cohort was heterogenous in nature and the study did not evaluate rectal cancer only, results were similar to the present study findings, despite a slightly different type of exposure. To our knowledge, only one other report has studied mGPS and the risk for anastomotic leakage after anterior resection due to rectal cancer. This small study identified mGPS = 2, as compared to mGPS = 0 or mGPS = 1, as an independent predictor for anastomotic leakage [10] with a very high point estimate and wide confidence intervals; the present study confirms this result, although with a lower estimate. In all likelihood, this is explained by the larger sample size in our study and another analytical approach, emphasising a causal interpretation. A few other studies have reported that the mGPS score and equivalent indices may be associated with postoperative complications in general [31, 32] after colorectal cancer surgery, further corroborating the findings in the present study. Similarly, there is a paucity of data concerning preoperative CAR and the subsequent risk of leakage. In a colorectal cancer cohort, comprising a similar number of anterior resections as in the present study, a CAR higher than 2.44 was found to be an independent risk factor for leakage [8]. Although CAR was shown to be an important predictor in the present study as well, differences such as no defunctioning stoma use and a markedly higher CAR cut-off make comparisons difficult, not the least as information on neoadjuvant treatment was missing from the above report.

### Mechanisms

The inflammatory response to a tumour is linked to malnutrition and a decline in immunological response [32]. Importantly, the inflammation process leads to reduced albumin levels, regardless of the nutritional state. CRP is another acute phase protein, with a more immediate response to inflammation; as such, the combination of these inflammation-induced proteins might convey a more complete picture of the inflammatory state prior to surgery. The pathogenesis of anastomotic leakage is not fully understood, although it is recognized that leaks in almost all cases seem to be a multifactorial failure of wound healing rather than due to technical error [33]. Preoperative inflammation is associated with a higher incidence of postoperative infection, such as anastomotic leakage [34]. Potentially, preoperatively established systemic inflammation and changes in the local intestinal environment may persist postoperatively and influence the healing process [35] and could thus not only act as a marker for leakage, but also induce it.

## Conclusion

A high degree of preoperative inflammation seems to be involved in the development of anastomotic leakage after anterior resection for cancer. However, inclusion of mGPS and CAR into prediction models with available clinical data did not result in accurate leakage prediction. Further research to delineate the role of preoperative inflammation is needed, as more precise modelling could facilitate clinical decision-making, potentially reducing the use of temporary stomas in low-risk patients and avoiding an anastomosis altogether in high-risk patients.

### Supplementary Information

Below is the link to the electronic supplementary material.

Supplementary file1 (PDF 148 KB)

## Data Availability

Upon reasonable request, data and methodology can be shared. This also applies to the registry-based data used in the current study, while access to such data might be subject to external review by the Swedish Colorectal Cancer Registry steering committee.

## Declarations

### Permission to reproduce material from other sources

No material from other sources was included in this article.
