# Supplementary material for: The modified Glasgow Prognostic Score indicates an increased risk of anastomotic leakage after anterior resection for rectal cancer
Source: Int J Colorectal Dis. 2023 Jul 20;38(1):200. doi: 10.1007/s00384-023-04496-5 (PMC10359376; doi:10.1007/s00384-023-04496-5)
Supplement: Supplementary file 1 — Supplementary file1 (PDF 148 KB) [file 384_2023_4496_MOESM1_ESM.pdf]

Supplementary Tables

Table S1. Clinical and demographic data for *n* = 418 patients (patients with missing on preoperative C-reactive protein and preoperative albumin are excluded; patients with missing covariate values are included), stratified on C-reactive protein-to-albumin ratio (CAR). CAR is categorized into two categories using an estimated optimal cut-off point. Fisher's exact test for testing independence between anastomotic leakage and CAR resulted in p-value = 0.006.

	**CAR ≤ 0.36 (N=365)**	**CAR > 0.36 (N=53)**	**Overall (N=418)**
**Age**			
Median (IQR)	66 (58–72)	70 (62–75)	66 (59–72)
**Sex**			
Female	145 (39.7%)	18 (34.0%)	163 (39.0%)
Male	220 (60.3%)	35 (66.0%)	255 (61.0%)
**Charlson Comorbidity Index**			
0	257 (70.4%)	35 (66.0%)	292 (69.9%)
1	63 (17.3%)	8 (15.1%)	71 (17.0%)
2	29 (7.9%)	5 (9.4%)	34 (8.1%)
≥3	16 (4.4%)	5 (9.4%)	21 (5.0%)
**ASA fitness grade**			
I	83 (22.7%)	7 (13.2%)	90 (21.5%)
II	227 (62.2%)	36 (67.9%)	263 (62.9%)
III–V	51 (14.0%)	10 (18.9%)	61 (14.6%)
Missing	4 (1.1%)	0 (0%)	4 (1.0%)
**Smoker**			
No	322 (88.2%)	43 (81.1%)	365 (87.3%)
Yes	24 (6.6%)	4 (7.5%)	28 (6.7%)
Missing	19 (5.2%)	6 (11.3%)	25 (6.0%)
**Body Mass Index (kg/m^2)**			
Median (IQR)	25.4 (23.1–28.4)	25.5 (24.2–27.7)	25.4 (23.2–28.2)
Missing	6 (1.6%)	0 (0%)	6 (1.4%)
**Tumour height (cm)**			
Median (IQR)	10 (9–12)	11 (9–12)	10 (9–12)
Missing	0 (0%)	1 (1.9%)	1 (0.2%)
**Clinical T stage**			
cT1-2	148 (40.5%)	10 (18.9%)	158 (37.8%)
cT3	178 (48.8%)	32 (60.4%)	210 (50.2%)
cT4	28 (7.7%)	10 (18.9%)	38 (9.1%)

Table S1. Clinical and demographic data for *n* = 418 patients (patients with missing on preoperative C-reactive protein and preoperative albumin are excluded; patients with missing covariate values are included), stratified on C-reactive protein-to-albumin ratio (CAR). CAR is categorized into two categories using an estimated optimal cut-off point. Fisher's exact test for testing independence between anastomotic leakage and CAR resulted in p-value = 0.006.

	**CAR ≤ 0.36 (N=365)**	**CAR > 0.36 (N=53)**	**Overall (N=418)**
cTX	11 (3.0%)	1 (1.9%)	12 (2.9%)
**Clinical N stage**			
cN0	187 (51.2%)	15 (28.3%)	202 (48.3%)
cN1-2	166 (45.5%)	37 (69.8%)	203 (48.6%)
cNX	12 (3.3%)	1 (1.9%)	13 (3.1%)
**Clinical M stage**			
cM0	345 (94.5%)	51 (96.2%)	396 (94.7%)
cM1	20 (5.5%)	2 (3.8%)	22 (5.3%)
**Neoadjuvant therapy**			
No	182 (49.9%)	18 (34.0%)	200 (47.8%)
Radiotherapy	139 (38.1%)	23 (43.4%)	162 (38.8%)
Radiochemotherapy	44 (12.1%)	12 (22.6%)	56 (13.4%)
**Surgical technique**			
Open	130 (35.6%)	22 (41.5%)	152 (36.4%)
Minimally invasive	235 (64.4%)	31 (58.5%)	266 (63.6%)
**Type of mesorectal excision**			
Total	277 (75.9%)	39 (73.6%)	316 (75.6%)
Partial	88 (24.1%)	14 (26.4%)	102 (24.4%)
**Defunctioning stoma**			
No	71 (19.5%)	10 (18.9%)	81 (19.4%)
Yes	294 (80.5%)	43 (81.1%)	337 (80.6%)
**Perioperative bleeding (ml)**			
Median (IQR)	100 (50–300)	200 (99–500)	100 (50—350)
Missing	10 (2.7%)	0 (0%)	10 (2.4%)

IQR = interquartile range; ASA = American Society of Anesthesiologists

TABLE S2. Prediction of anastomotic leakage within 12 months. Association between preoperative modified Glasgow Prognostic Score (mGPS) and anastomotic leakage within 12 months. Logistic regression (based on 10 imputed datasets) and n = 418 patients. **Covariate set:** *Logistic regression including all predictors*: mGPS, age, sex, Body Mass Index, clinical T stage, clinical N stage, clinical M stage, Charlson comorbidity index group (CCI), current smoking status, neoadjuvant therapy, American Society of Anesthesiologists' (ASA) fitness grade, type of mesorectal excision, defunctioning stoma, previous surgery, hospital volume and minimally invasive surgery; *Logistic regression including predictors selected by lasso variable selection (in at least one of the 10 imputed datasets)*: mGPS (selected in 10 datasets), sex (1), clinical T stage (1), clinical N stage (10), Charlson comorbidity index group (2), current smoking status (3), neoadjuvant therapy (10), ASA (10), type of mesorectal excision (10), hospital volume (10).

	**OR (95% CI)**	**p-value**
	Logistic regression including all predictors	
**mGPS = 1**	1.48 (0.64–3.44)	0.36
**mGPS = 2**	2.99 (1.01–8.85)	0.05
**Age**	1.00 (0.97–1.03)	0.79
**Male sex**	1.33 (0.72–2.44)	0.36
**Body mass index**	1.00 (0.93–1.08)	0.98
**cT3**	0.66 (0.33–1.34)	0.25
**cT4**	0.97 (0.33–2.89)	0.96
**cTX**	0.42 (0.04–4.51)	0.47
**cN1**	1.72 (0.84–3.54)	0.14
**cNX**	4.16 (0.63–27.59)	0.14
**cM1**	1.97 (0.62–6.30)	0.25
**Smoker**	1.90 (0.65–5.55)	0.24
**CCI = 1**	0.96 (0.43–2.17)	0.93
**CCI = 2**	1.87 (0.69–5.09)	0.22
**CCI ≥ 3**	0.67 (0.15–3.09)	0.61
**Radiotherapy**	1.70 (0.77–3.75)	0.19
**Chemoradiotherapy**	1.26 (0.40–3.97)	0.69
**Total mesorectal excision**	5.09 (1.62–15.97)	**<0.01**
**Defunctioning stoma**	0.41 (0.14–1.16)	0.09
**ASA = II**	1.22 (0.56–2.67)	0.61
**ASA = III–IV**	2.05 (0.71–5.93)	0.19
**Minimally invasive surgery**	0.99 (0.55–1.80)	0.98
**Hospital volume**	**1.03 (1.01–1.05)**	**<0.01**
**Previous surgery**	1.05 (0.57–1.95)	0.87
	Logistic regression including predictors selected by lasso variable selection	
**mGPS = 1**	1.42 (0.62–3.21)	0.41
**mGPS = 2**	2.98 (1.01–8.82)	0.05
**Male sex**	1.32 (0.75–2.34)	0.34
**cT3**	0.65 (0.32–1.32)	0.23
**cT4**	0.93 (0.32–2.73)	0.90
**cTX**	0.40 (0.04–4.32)	0.45
**cN1**	1.82 (0.90–3.70)	0.10
**cNX**	4.11 (0.61–27.41)	0.15
**Smoker**	1.84 (0.67–5.06)	0.24
**CCI = 1**	1.02 (0.47–2.25)	0.96
**CCI = 2**	1.83 (0.69–4.86)	0.23
**CCI ≥ 3**	0.67 (0.15–3.04)	0.60
**Radiotherapy**	1.62 (0.74–3.56)	0.23
**Chemoradiotherapy**	1.33 (0.43–4.05)	0.62
**Total mesorectal excision**	2.64 (1.16–6.02)	**0.02**

**ASA = II**	1.28 (0.62–2.66)	0.50
**ASA = III–IV**	2.29 (0.84–6.28)	0.11
**Hospital volume**	**1.03 (1.01–1.05)**	**<0.01**

TABLE S3. Prediction of anastomotic leakage within 12 months. Association between preoperative C-reactive protein-to-albumin ratio (CAR) and anastomotic leakage within 12 months. Logistic regression (based on 10 imputed datasets) and n = 418 patients. **Covariate set:** *Logistic regression including all predictors*: CAR, age, sex, Body Mass Index, clinical T stage, clinical N stage, clinical M stage, Charlson comorbidity index group, current smoking status, neoadjuvant therapy, American Society of Anesthesiologists' (ASA) fitness grade, type of mesorectal excision, defunctioning stoma, previous surgery, hospital volume and laparoscopy; *Logistic regression including predictors selected by lasso variable selection (in at least one of the 10 imputed datasets)*: CAR (selected in 10 datasets), sex (4), clinical T stage (3), clinical N stage (10), clinical M stage (2), Charlson comorbidity index group (4), current smoking status (4), neoadjuvant therapy (10), ASA (10), type of mesorectal excision (10), hospital volume (10). CAR is categorized into two categories using an estimated optimal cut point.

	**OR (95% CI)**	**p-value**
	Logistic regression including all predictors	
**CAR > 0.36**	2.41 (1.16–5.00)	**0.02**
**Age**	1.00 (0.97–1.03)	0.84
**Male sex**	1.34 (0.73–2.47)	0.35
**Body mass index**	1.00 (0.93–1.08)	1.00
**cT3**	0.66 (0.33–1.33)	0.25
**cT4**	1.01 (0.34–3.01)	0.98
**cTX**	0.38 (0.04–4.12)	0.43
**cN1**	1.65 (0.80–3.39)	0.18
**cNX**	4.42 (0.67–29.01)	0.12
**cM1**	2.12 (0.65–6.90)	0.21
**Smoker**	2.12 (0.70–6.45)	0.19
**CCI = 1**	0.97 (0.43–2.21)	0.95
**CCI = 2**	1.79 (0.65–4.87)	0.26
**CCI ≥ 3**	0.62 (0.13–2.84)	0.54
**Radiotherapy**	1.73 (0.78–3.81)	0.18
**Chemoradiotherapy**	1.22 (0.39–3.81)	0.73
**Total mesorectal excision**	5.39 (1.73–16.78)	**<0.01**
**Defunctioning stoma**	0.39 (0.14–1.11)	0.08
**ASA = II**	1.22 (0.56–2.67)	0.62
**ASA = III–IV**	2.30 (0.80–6.63)	0.12
**Minimally invasive surgery**	1.03 (0.56–1.87)	0.94
**Hospital volume**	**1.03 (1.01–1.05)**	**<0.01**
**Previous surgery**	1.09 (0.59–2.02)	0.79
	Logistic regression including predictors selected by lasso variable selection	
**CAR > 0.36**	2.32 (1.13–4.78)	**0.02**
**Male sex**	1.35 (0.76–2.39)	0.31
**cT3**	0.65 (0.32–1.30)	0.22
**cT4**	0.95 (0.33–2.79)	0.93
**cTX**	0.37 (0.03–4.03)	0.42
**cN1**	1.76 (0.86–3.59)	0.12
**cNX**	4.44 (0.67–29.43)	0.12
**cM1**	1.99 (0.63–6.32)	0.24
**Smoker**	2.12 (0.74–6.04)	0.16
**CCI = 1**	1.04 (0.47–2.31)	0.92
**CCI = 2**	1.77 (0.66–4.72)	0.25
**CCI ≥ 3**	0.64 (0.14–2.88)	0.56
**Radiotherapy**	1.65 (0.75 –.63)	0.21

**Chemoradiotherapy**	1.18 (0.39–3.58)	0.77
**Total mesorectal excision**	2.89 (1.25–6.66)	**0.01**
**ASA = II**	1.22 (0.59–2.55)	0.59
**ASA = III–IV**	2.50 (0.92–6.79)	0.07
**Hospital volume**	**1.03 (1.01–1.05)**	**<0.01**
